# Public school teachers' occupational stress across different school types: a nationwide survey during the prolonged COVID-19 pandemic in Japan

**DOI:** 10.3389/fpubh.2023.1287893

**Published:** 2023-10-19

**Authors:** Kenjiro Tsubono, Sachiko Mitoku

**Affiliations:** ^1^Department of Psychosomatic Medicine, Tokai Central Hospital, Gifu, Japan; ^2^Department of Health Management, Tokai Central Hospital, Gifu, Japan

**Keywords:** COVID-19 pandemic, interpersonal conflicts, public schools, school types, stressors, stress responses, teachers, working hours

## Abstract

**Objectives:**

School teaching is regarded as one of the most stressful professions worldwide. To maintain schoolteachers' mental health, the factors influencing occupational stress among schoolteachers must be clarified. This study aimed to investigate public school teachers' work-related stress considering the differences in school types using data from a large-scale nationwide survey conducted during the prolonged coronavirus disease 2019 (COVID-19) pandemic in Japan.

**Methods:**

Data from a nationwide survey of public school teachers performed between June 2019 and December 2022 were analyzed. The dataset consisted of repeated cross-sectional data. The total number of participants was 270,777 in 2019, 296,599 in 2020, 299,237 in 2021, and 307,866 in 2022. Information on working hours, job demands, workplace support, stress response, and perceived main stressors were assessed for each type of public school.

**Results:**

Regardless of school type, quantitative workload and long working hours were the most significant factors affecting teachers' stress responses. However, stress-related factors among teachers varied significantly between school types. The percentage of junior high school teachers who perceived “extra-curricular club activities” as their main stressor was the highest among all school types. The highest proportion of elementary school teachers perceived “dealing with difficult students” as their main stressor. Meanwhile, interpersonal conflict scores were the highest among special needs school teachers. Teachers' workload and stress levels significantly increased in the third year of the COVID-19 pandemic (2022) compared to the pre-pandemic year (2019) in all school types despite the marginally small score differences.

**Conclusions:**

This study highlighted the importance of reducing teachers' workload for their mental health regardless of school types. Meanwhile, perceived work-related stress among teachers differed significantly between school types. Given the possible prolonged impacts of the pandemic on teachers' occupational stress, teachers' stress levels must be monitored throughout and after the pandemic. The results suggest that increasing the number of schoolteachers and support staff and providing adequate organizational support are necessary to prevent teachers' sick leave due to mental disorders. In addition, taking comprehensive countermeasures against teachers' occupational stress, considering the differences in school types, is crucial for safeguarding schoolteachers' mental health.

## 1. Introduction

Teaching is one of the most stressful professions worldwide (1, 2). A high prevalence of mental disorders, such as depression and anxiety, have been identified among schoolteachers (3, 4). Consequently, teachers exhibit relatively high levels of stress-related symptoms and low levels of mental wellbeing compared with other occupations (5, 6). Burnout significantly contributes to teacher attrition (7). School teaching is one of the professions with the highest burnout rate (8). Teachers' work-related stress is associated with decreased job performance and increased burnout, which eventually affects their professional accomplishments (9). High levels of occupational stress among schoolteachers negatively affect individuals and society (10).

Schoolteachers are exposed to various sources of work-related stress. One of the major stressors among teachers is students' misbehavior (11). Moreover, interpersonal conflicts among co-workers are positively related to burnout rates among teachers (12, 13). In addition, long working hours among schoolteachers is a major social issue globally (14, 15) and is significantly associated with stress-related disorders among teachers (15, 16). In addition to teaching duties, teachers are burdened with multiple administrative and clerical tasks (17). According to the Teaching and Learning International Survey performed in 2018 (TALIS, 2018), teachers experience higher levels of stress in their school management duties or administrative work than in their classroom teaching tasks (18).

Schoolteachers' occupational stress varies depending on the school setting. Some studies indicate that primary school teachers experience greater stress and burnout than high school teachers (19–21). Timms et al. (19) indicated that a gender ratio imbalance and high job stress among female teachers could be the main reasons for the increased stress levels of primary school teachers (19). Generally, primary school students require more support because of their immaturity (22). Accordingly, teachers may be devoting more time and effort to primary school students, which may explain elevated stress levels among primary school teachers (23). Conversely, other studies have demonstrated that secondary school teachers are more stressed than primary school teachers (24, 25). Kavita et al. (24) analyzed seven stress factors: relationship with parents and co-workers, workload, time pressure, student attitude, workplace support, and lack of resources. Regarding all these stress factors, secondary school teachers experienced more stress than primary school teachers (24). Kongcharoen et al. (25) reported that secondary school teachers experienced higher overall stress than primary school teachers due to financial challenges and various work obligations (25). Studies on stress among special education teachers (teachers who work with students with learning or cognitive difficulties) unveiled that they experienced substantial work-related stress (26–28). Special education teachers struggled with inadequate training opportunities (27), lack of support from the organization and administration (26), and the perception that students are not excelling academically despite their efforts (28). Thus, considerable differences in schoolteachers' stress structures may exist between different school types.

In Japan, leaves of absence among schoolteachers due to mental health problems have become an urgent social concern. The percentage of schoolteachers on leave due to mental disorders has increased by approximately sixfold from 0.11% in 1992 to 0.64% in 2021 (29). In addition to their essential teaching tasks, teachers in Japan are tasked with various duties, such as related clerical work, school management, parent-teacher association activities, and extra-curricular club activities. The TALIS 2018 demonstrated that the working hours of schoolteachers in Japan were the longest among the OECD participating countries (18).

Public education has played a vital role in Japanese society. Public schools account for 96% of all primary and lower secondary schools in Japan (30). Mainly, Japan has four types of public school (excluding higher education institutions) according to education levels and the presence of students with physical or learning disabilities: elementary schools, junior high schools, high schools, and special needs schools. Elementary schools comprise 6-year education programs in which children's school attendance typically starts at the age of six. After graduating from elementary schools, students enroll in junior high schools which comprise 3-year lower secondary education programs. Compulsory education begins with 6 years of elementary school and ends with 3 years of junior high school. After completing a 9-year compulsory education, most students proceed to high schools which are normally attended for 3 years between the ages of 15 and 18 years. Special needs schools, which are divided into four educational levels (kindergarten, elementary, lower, and upper secondary), are for children with comparatively severe physical or learning disabilities. Students with mild disabilities attending regular elementary and junior high schools also receive special needs education.

Junior high school teachers in Japan tend to work extremely long hours (18). In junior high schools in Japan, extra-curricular club activities are enthusiastically pursued, with many teachers serving as advisors or coordinators (31). Junior high school teachers spend an average of 7.6 h a week on extra-curricular club activities, whereas elementary school teachers only spend 0.6 h a week (32). These activities and related club tournaments generally occur after school or on weekends, compelling teachers to extend their working hours or report to work on weekends and holidays. According to a survey conducted by the Ministry of Education, Culture, Sports, Science and Technology (MEXT), the working hours of junior high school teachers were the longest among the three types of public schools in Japan (elementary, junior high, and high schools) (32). Approximately 60% of junior high school teachers worked 60 h a week or more (32). Thus, many engage in overtime work of over 80 h a month, which is considered a criterion for sudden death from overwork due to the increased risk of cardiovascular disease (33).

In Japan, special needs school teachers experience marked occupational stress due to the discrepancies between teachers' needs and national educational policies (34), similar to the conditions among special education teachers in other nations (26, 27). They also face inadequate organizational support for their particular working conditions (34). Conflicts among co-workers have also been linked to teachers' burnout, which is directly associated with sick leave due to mental illness (12). Team teaching, commonly employed in special needs schools in Japan, has been associated with teachers' stress reactions, mainly because teachers with different teaching philosophies are compelled to collaborate (34). For these various reasons, a survey conducted by MEXT in 2021 indicated that the percentage of teachers leaving a job due to mental health problems was the highest in special needs schools among all types of public schools in Japan (29).

It is clear then that teachers' stress levels and related factors vary by school setting. To take comprehensive countermeasures against increased sick leave among teachers due to mental illness, the factors contributing to teachers' work-related stress, considering the differences in school types, must be clarified. However, the influence of school type on teachers' stress has not been adequately considered in a national survey with a sufficiently high participation from the target population.

In Japan, the government implemented the Stress Check Program in 2015, to mitigate workers' sick leave due to mental disorders (35). The program must be executed once a year in workplaces with 50 or more employees (35). In this program, workers' job stressors, and stress levels are examined. Every year, a significant number of public school teachers across Japan have participated in this program.

This study sought to examine schoolteachers' occupational stress and clarify the stress factors by considering the differences in school type using large-scale nationwide survey data. Finally, the study aimed to offer a useful proposal for protecting teachers' mental health. Data from the Stress Check Program, which is conducted on numerous public school teachers across Japan, were analyzed.

Based on the context described above, we present the following research hypotheses:

Hypothesis 1: Regardless of school type, quantitative workload and long working hours are the most significant factors affecting teachers' stress responses.

Hypothesis 2: Stress response scores among teachers in junior high and special needs schools are the highest among all types of public schools.

Hypothesis 3: Interpersonal conflict scores among teachers in special needs schools are higher than those in any other type of public school.

The COVID-19 pandemic has caused significant global challenges for schoolteachers worldwide (36). During the pandemic, a substantial prevalence of anxiety and depression among teachers has been observed (36, 37). Teachers experience high levels of stress as a result of workloads involving unfamiliar online instruction and the implementation of countermeasures against the spread of infection while performing routine school duties (36). In Japan, mild lockdowns have been intermittently implemented because the pandemic has showed a repeated pattern of expansion and contraction. The government maintained the classification level of COVID-19 as Category II under the Infectious Disease Control Law until May 2023, which required people to take strict countermeasures against the spread of infection for a total of 3 years (38). This situation holds true for school workplaces, where strict infection control measures have been implemented for a considerably long period. Teachers' stress levels are expected to increase significantly during this prolonged pandemic period. Thus, we propose the fourth hypothesis:

Hypothesis 4: Teachers' (quantitative and qualitative) workloads and stress levels have significantly increased during the prolonged COVID-19 pandemic.

## 2. Materials and methods

### 2.1. Sample and data collection procedure

We used data from the Stress Check Program performed by the Mutual Aid Association of Public School Teachers for public school (primary, secondary, and special needs schools) employees in participating educational institutions across Japan. The number of eligible public school employees for this program was approximately 350,000 per year. The survey is conducted yearly between June and December through an online questionnaire. The survey did not include questions specifically regarding the impact of the COVID-19 pandemic on schoolteachers' stress; nonetheless, it did include various questions concerning teachers' work-related stress, such as job workload, stress responses, working hours, and perceived main stressors. The total numbers of public school employees completing this questionnaire were 270,777 in 2019, 296,599 in 2020, 299,237 in 2021, and 307,866 in 2022, which comprised 80.0%, 81.1%, 82.9%, and 82.3% of all eligible employees, respectively. We could not acquire precise information relating to the proportion of public school teachers who underwent this “Stress Check” examination in all 4 years from 2019 to 2022. However, considering the program's high response rate (80.0–82.9%), a substantial number of public school teachers are most likely to have completed the examination in all 4 years.

The inclusion criteria for participating were as follows: (1) a full-time public school teacher (working at elementary, junior high, high, and special needs schools). The exclusion criteria were as follows: (1) a part-time teacher, (2) a teacher with administrative positions (a school principal and a vice-principal), (3) a nursing teacher (responsible for offering first aid to sick or injured school children), (4) a nutrition teacher (responsible for providing a nutrition education program), and (5) a clerical worker. No participants had missing data. The total number of eligible participants was 205,255 in 2019, 224,347 in 2020, 226,506 in 2021, and 232,577 in 2022. Table 1 exhibits the demographic characteristics of the participants.

**Table 1:** Participants' demographics.

		**2019**		**2020**		**2021**		**2022**	
		***n***	**%**	***n***	**%**	***n***	**%**	***n***	**%**
**Elementary school**	Men	34,569	37.0%	38,388	37.0%	39,296	37.2%	41,077	37.2%
	Women	58,984	63.0%	65,341	63.0%	66,262	62.8%	69,228	62.8%
	Total	93,553	100.0%	103,729	100.0%	105,558	100.0%	110,305	100.0%
	**Age**								
	≤ 29	22,018	23.5%	24,081	23.2%	24,226	23.0%	25,212	22.9%
	30–39	21,224	22.7%	23,688	22.8%	24,661	23.4%	26,102	23.7%
	40–49	19,967	21.3%	22,080	21.3%	22,481	21.3%	23,465	21.3%
	50–59	23,491	25.1%	25,122	24.2%	24,714	23.4%	24,737	22.4%
	≥60	6,853	7.3%	8,758	8.4%	9,476	9.0%	10,789	9.8%
**Junior high school**	Men	30,110	57.2%	33,725	57.0%	34,143	56.8%	35,347	56.6%
	Women	22,574	42.8%	25,479	43.0%	25,981	43.2%	27,112	43.4%
	Total	52,684	100.0%	59,204	100.0%	60,124	100.0%	62,459	100.0%
	**Age**								
	≤ 29	11,403	21.6%	12,612	21.3%	12,881	21.4%	13,204	21.1%
	30-39	13,442	25.5%	15,318	25.9%	15,660	26.0%	16,754	26.8%
	40-49	11,729	22.3%	12,961	21.9%	12,889	21.4%	13,024	20.9%
	50-59	12,548	23.8%	13,591	23.0%	13,286	22.1%	13,303	21.3%
	≥60	3,562	6.8%	4,722	8.0%	5,408	9.0%	6,174	9.9%
**High school**	Men	27,040	66.7%	28,067	66.2%	27,480	65.9%	26,880	65.8%
	Women	13,768	33.7%	14,353	33.8%	14,249	34.1%	13,963	34.2%
	Total	40,808	100.0%	42,420	100.0%	41,729	100.0%	40,843	100.0%
	**Age**								
	≤ 29	5,221	12.8%	5,430	12.8%	5,191	12.4%	5,165	12.6%
	30-39	8,306	20.4%	8,672	20.4%	8,634	20.7%	8,624	21.1%
	40-49	11,394	27.9%	11,489	27.1%	10,831	26.0%	10,123	24.8%
	50-59	12,334	30.2%	12,612	29.7%	12,287	29.4%	11,678	28.6%
	≥60	3,553	8.7%	4,217	9.9%	4,786	11.5%	5,253	12.9%
**Special needs school**	Men	7,023	38.6%	7,350	38.7%	7,300	38.2%	7,165	37.8%
	Women	11,187	61.4%	11,644	61.3%	11,795	61.8%	11,805	62.2%
	Total	18,210	100.0%	18,994	100.0%	19,095	100.0%	18,970	100.0%
	**Age**								
	≤ 29	3,267	17.9%	3,301	17.4%	3,172	16.6%	3,058	16.1%
	30-39	3,994	21.9%	4,247	22.4%	4,417	23.1%	4,492	23.7%
	40-49	5,098	28.0%	5,132	27.0%	5,065	26.5%	4,920	25.9%
	50-59	4,885	26.8%	5,132	27.0%	5,081	26.6%	5,002	26.4%
	≥60	966	5.3%	1,182	6.2%	1,360	7.1%	1,498	7.9%

### 2.2. Measurements

#### 2.2.1. Working hours

We collected data on working hours per day with seven answer options as follows: (1) <8 h, (2) 8 to 9 h, (3) 9 to 10 h, (4) 10 to 11 h, (5) 11 to 12 h, (6) 12 to 13 h, and (7) 13 h or more. The data on working hours in the survey were based on self-reported information, including the time spent on various school duties other than educational tasks. These included school management duties, clerical work, extracurricular club activities, and parental contact.

#### 2.2.2. Brief job stress questionnaire

In this study, the Brief Job Stress Questionnaire (BJSQ) was used to assess schoolteachers' work-related stress. Several language versions of the BJSQ are available (39). The BJSQ is an established stress scale used to identify high-stress workers, and is broadly used in occupational health in Japan (40, 41). The BJSQ was developed in reference to the Generic Job Stress Questionnaire designed by the United States of America National Institute for Occupational Safety and Health (42). The BJSQ was also formulated based on the Job Demand-Control-Support model, the central hypothesis of which is that combinations of job demand, job control, and social support are associated with workers' stress levels (43). The scale comprises 57-items and assesses three aspects of work-related stressors: job demands (17 items), stress responses (29 items), and social support factors (11 items). Among job demands, the BJSQ includes quantitative workload (three items; e.g., “I have an extremely large amount of work to do”), qualitative workload (three items; e.g., “I have to pay very careful attention”), physical demands (one item; “My job requires a lot of physical work”), job control (three items; e.g., “I can work at my own pace”), skill utilization (one item; “My knowledge and skills are used at work”), interpersonal conflict (three items; e.g., “There are differences of opinion within my department”), poor physical environment [one item; “My working environment is poor (e.g. noise, lighting, temperature, ventilation”)], suitable jobs (one item; “This job suits me well”), and meaningfulness of work (one item; “My job is worth doing”). Stress responses include vigor (three items; e.g., “I have been very active”), anger-irritability (three items; e.g., “I have felt angry”), fatigue (three items; e.g., “I have felt exhausted”), anxiety (three items; e.g., “I have felt restless”), depression (six items; e.g., “I have felt gloomy”), and physical symptoms (11 items; e.g., “I have experienced headaches”). Social support factors include support from supervisors (three items; e.g., “How freely can you talk with your supervisors?”), co-workers (three items), and family and friends (three items). Each item was rated on a four-point Likert scale (1 = almost never, 2 = sometimes, 3 = often, and 4 = almost always). The stress response scores range from 29 to 116, with higher scores meaning higher stress levels. The scores on the three-item scale range from 3 to 12 (the scores on the one-item scale range from one to four). Higher scores indicate higher levels of stress for quantitative and qualitative workloads, physical demands, interpersonal conflict, and poor physical environment. Higher scores indicate better work conditions for job control, skill utilization, suitable jobs, and meaningfulness of work. Regarding the social support factors, higher scores indicate higher levels of social support.

The reliability and validity of this questionnaire are well established (44). All BJSQ scales presented acceptable alpha coefficients (e.g., quantitative workload, 0.82; qualitative workload, 0.73; job control, 0.76; stress responses, 0.90) (44, 45). Stress response scores measured using the BJSQ successfully predict the occurrence of depression among employees (45). The BJSQ has been used to evaluate work-related stressors and stress levels in various professions, such as schoolteachers, healthcare professionals, and firefighters (46–49).

#### 2.2.3. Perceived main stressors of teachers

Participants were asked to select their main stressors out of the following 12 items (up to two items can be selected): (1) responsibility for students' learning, (2) school management duties, (3) providing a demonstration lesson, (4) managing extra-curricular club activities (5) dealing with difficult students, (6) dealing with challenging parents, (7) workload of clerical tasks, (8) relationship with co-workers, (9) relationship with supervisors, (10) unfamiliar work environment (due to a transfer), (11) long commuting time, and (12) personal problems. The survey items on schoolteachers' main stressors were chosen by the Mutual Aid Association of Public School Teachers based on the opinions of mental health professionals such as psychiatrists and psychologists in affiliated organizations. This study investigated the main stressors experienced by teachers in each type of public school.

### 2.3. Statistical analysis

Continuous variables are presented as means (*M*) with standard deviation (*SD*), and categorical variables presented as the number of cases with percentages. Differences in continuous variables were compared using Welch's one-way ANOVA, and a *post-hoc* analysis was performed using the Games–Howell test. Eta-squared (η^2^) was calculated as the effect size for ANOVA using 0.01, 0.06, and 0.14 considered small, medium, and large effect sizes (50). Accordingly, we interpreted the eta-squared value of 0.01 as the minimum threshold of practical significance.

A multiple linear regression analysis was performed to assess the relationship between each scale of the BJSQ and stress responses after adjusting for gender and years of experience as a teacher for each school type. We also examined whether the size of each regression coefficient differed statistically between different school types. This procedure was performed by adding the interaction term between school type (after creating dummy variables) and each predictor variable to the regression equation, as well as examining its statistical significance (51).

To assess the multicollinearity between variables, we first examined the correlation coefficients for each pair of predictor variables. If the correlation coefficients for two variables were 0.8 or above, only one was used in the analysis. Multicollinearity was evaluated using variance inflation factors (VIF). We regarded a VIF exceeding 5.0 as indicating the presence of multicollinearity. We did not examine the interactions between the predictor variables in this study.

Cross-tabulated frequencies and percentages were calculated for the statistical analysis of categorical variables. A chi-squared test was performed to assess the association between categorical valuables. Cramer's V was used to calculate the effect size of the test. Conventionally, Cramer's V values of < 0.1 was considered negligible; 0.1, a small effect; 0.3, a medium effect; and 0.5, a large effect (33). Accordingly, we regarded the effect size value of 0.1 as the minimum threshold of practical significance. All the statistical analyses were conducted using SPSS version 28 (IBM Corp., Armonk, NY, USA). The level of significance for each test was set at *p* < 0.05.

### 2.4. Ethical considerations

The study was performed in accordance with the latest version of the Declaration of Helsinki, and was approved by the Institutional Review Board of Tokai Central Hospital (Reference no. 2022082601). This study used existing data, which were already completely anonymized and untraceable. The ethics committee of the hospital confirmed that all procedures were conducted appropriately and concluded that informed consent was not necessary for this study.

## 3. Results

### 3.1. Participants' characteristics

Participants' demographics are presented in Table 1. The largest number of teachers were elementary school teachers (*N* = 93,553–110,305 per year), followed by junior high school teachers (*N* = 52,684–62,459 per year). For elementary and special needs schools, the proportion of women was higher than that of men (62.8–63.0% and 61.3–62.2%, respectively).

### 3.2. Comparisons of working hours by school types

**Figure 1:**
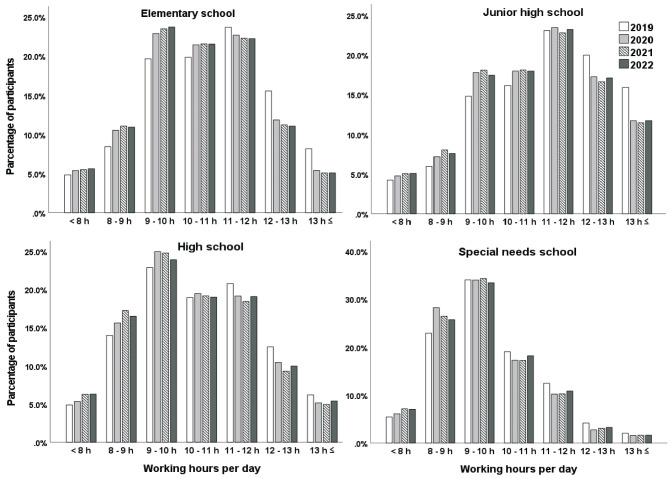
Comparisons of school teachers' working hours per day from 2019 to 2022 by school types.

Figure 1 exhibits teachers' working hours per day for each school type. In the longest working-hour groups (11–12 h, 12–13 h, ≥13 h), the percentages of junior high school teachers were the highest (50.8–59.0%), followed by elementary school teachers (38.3–47.3%). Meanwhile, in the shortest working hour groups (< 8 h, 8–9 h 9–10 h), the percentage of special needs school teachers was the highest (62.3–68.2%). In all school types, the percentages of the longest working-hour groups (≥11 h) were the highest in 2019, and teachers' working hours significantly decreased after the pandemic began (2020–2022). The results of the chi-squared test demonstrated that the association between working hours and years was statistically significant (*p* < 0.001) in all school types; however, the effect sizes were marginally small (Cramer's V = 0.038–0.051).

### 3.3. Stress response scores in each working hour category from 2019 to 2022 by school types

**Figure 2:**
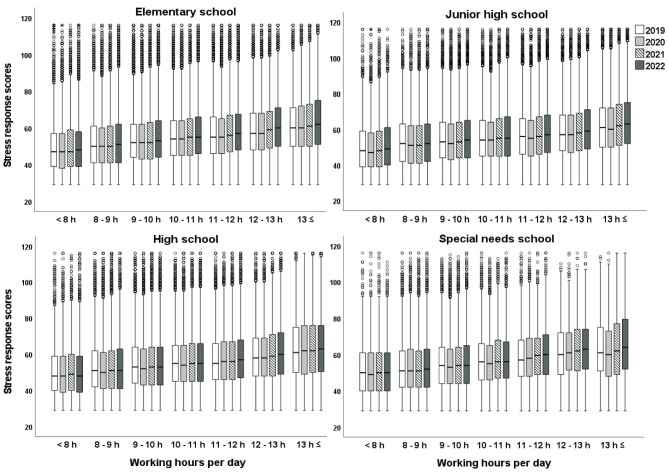
Stress response scores in each working hours category from 2019 to 2022 by school types.

Figure 2 presents box plots of the stress response scores in each working hour category from 2019 to 2022 according to school type. The results revealed that stress response scores significantly increased as working hours per day increased in all school types. Welch's ANOVA showed a significant difference in stress response scores between different working hour categories in all school types (*p* < 0.001, η^2^ = 0.027–0.043). In the same working hour category, stress response scores in 2022 were the highest in all school types, followed by those in 2021.

### 3.4. Participants' BJSQ scores from 2019 to 2022 by school types

Table 2 presents participants' BJSQ scores by school types between 2019 and 2022. Welch's ANOVA demonstrated a significant difference between school types in qualitative workload (η^2^ = 0.015–0.022), qualitative workload (η^2^ = 0.018–0.020), physical demands (η^2^ = 0.051–0.056), interpersonal conflict (η^2^ = 0.015–0.016), poor physical environment (η^2^ = 0.015–0.021), supervisor support (η^2^ = 0.014–0.018), and co-worker support (η^2^ = 0.014–0.017) in all four years (*p* < 0.001 for all the scales). The scores of quantitative and qualitative workloads were the highest among elementary school teachers, followed by those among junior high school teachers in all four years. The scores of interpersonal conflicts were the highest among special needs school teachers. The scores of supervisor and co-worker support were the highest among elementary school teachers, followed by those among junior high school teachers.

**Table 2:** Comparison of the BJSQ job stress and stress response scores among public school teachers between different school types.

		**2019**	**2020**	**2021**	**2022**	
		***M*** **(*****SD*****)**	***M*** **(*****SD*****)**	***M*** **(*****SD*****)**	***M*** **(*****SD*****)**	*p* ^a^
Quantitative workload	Elementary school	9.69 (1.91)	9.52 (1.94)	9.59 (1.94)	9.65 (1.94)	< 0.001
	Junior high school	9.59 (1.95)	9.43 (1.97)	9.54 (1.99)	9.62 (2.00)	< 0.001
	High school	9.07 (2.04)	8.96 (2.06)	9.03 (2.08)	9.11 (2.11)	< 0.001
	Special needs school	8.94 (1.94)	8.80 (1.94)	8.91 (1.98)	9.01 (1.99)	< 0.001
	Effect size^b^	0.022	0.017	0.017	0.015	
Qualitative workload	Elementary school	9.35 (1.72)	9.30 (1.74)	9.35 (1.73)	9.41 (1.73)	< 0.001
	Junior high school	9.06 (7.79)	9.01 (1.78)	9.06 (1.80)	9.14 (1.80)	< 0.001
	High school	8.68 (1.81)	8.67 (1.83)	8.69 (1.84)	8.73 (1.86)	< 0.001
	Special needs school	9.03 (1.74)	9.04 (1.75)	9.09 (1.76)	9.09 (1.76)	< 0.001
	Effect size^b^	0.020	0.018	0.018	0.019	
Physical demands	Elementary school	2.99 (0.75)	2.98 (0.75)	2.99 (0.75)	3.03 (0.75)	< 0.001
	Junior high school	2.80 (0.80)	2.80 (0.80)	2.80 (0.80)	2.84 (0.80)	< 0.001
	High school	2.49 (0.80)	2.50 (0.80)	2.51 (0.81)	2.53 (0.82)	< 0.001
	Special needs school	2.94 (0.78)	2.96 (0.78)	2.98 (0.79)	2.99 (0.78)	< 0.001
	Effect size^b^	0.056	0.051	0.052	0.053	
Interpersonal conflict	Elementary school	5.61 (1.82)	5.59 (1.83)	5.59 (1.82)	5.62 (1.82)	< 0.001
	Junior high school	6.08 (1.91)	6.06 (1.92)	6.06 (1.92)	6.11 (1.93)	< 0.001
	High school	6.06 (1.86)	5.99 (1.87)	6.01 (1.87)	6.01 (1.89)	< 0.001
	Special needs school	6.15 (1.76)	6.11 (1.77)	6.10 (1.77)	6.13 (1.77)	0.018
	Effect size^b^	0.016	0.015	0.015	0.016	
Poor physical environment	Elementary school	1.97 (0.84)	1.91 (0.81)	1.88 (0.80)	1.89 (0.80)	< 0.001
	Junior high school	2.07 (0.88)	2.02 (0.86)	2.00 (0.84)	2.02 (0.86)	< 0.001
	High school	2.23 (0.89)	2.18 (0.88)	2.17 (0.88)	2.18 (0.89)	< 0.001
	Special needs school	2.18 (0.86)	2.14 (0.85)	2.17 (0.86)	2.19 (0.87)	< 0.001
	Effect size^b^	0.015	0.016	0.021	0.020	
Job control	Elementary school	7.96 (1.80)	8.05 (1.78)	8.02 (1.80)	7.99 (1.81)	< 0.001
	Junior high school	7.84 (1.90)	7.91 (1.88)	7.86 (1.90)	7.81 (1.93)	< 0.001
	High school	8.03 (1.85)	8.10 (1.86)	8.06 (1.88)	8.05 (1.91)	< 0.001
	Special needs school	7.69 (1.81)	7.76 (1.81)	7.73 (1.83)	7.73 (1.83)	0.005
	Effect size^b^	0.003	0.003	0.003	0.003	
Skill utilization	Elementary school	3.20 (0.67)	3.22 (0.66)	3.21 (0.66)	3.20 (0.67)	< 0.001
	Junior high school	3.23 (0.70)	3.25 (0.70)	3.23 (0.70)	3.21 (0.71)	< 0.001
	High school	3.16 (0.72)	3.19 (0.72)	3.17 (0.72)	3.16 (0.72)	< 0.001
	Special needs school	3.03 (0.69)	3.06 (0.68)	3.05 (0.68)	3.04 (0.68)	0.002
	Effect size^b^	0.006	0.006	0.005	0.004	
Suitable jobs	Elementary school	3.03 (0.67)	3.05 (0.66)	3.02 (0.67)	3.01 (0.67)	< 0.001
	Junior high school	3.00 (0.70)	3.03 (0.68)	3.01 (0.70)	2.98 (0.70)	< 0.001
	High school	2.98 (0.69)	3.02 (0.69)	2.99 (0.69)	2.97 (0.70)	< 0.001
	Special needs school	2.99 (0.68)	3.01 (0.67)	2.99 (0.67)	2.98 (0.67)	< 0.001
	Effect size^b^	0.001	0.000	0.000	0.000	
Meaningfulness of work	Elementary school	3.36 (0.65)	3.36 (0.65)	3.32 (0.65)	3.28 (0.67)	< 0.001
	Junior high school	3.31 (0.68)	3.32 (0.67)	3.28 (0.69)	3.23 (0.70)	< 0.001
	High school	3.17 (0.71)	3.20 (0.70)	3.16 (0.71)	3.13 (0.72)	< 0.001
	Special needs school	3.27 (0.67)	3.29 (0.66)	3.25 (0.67)	3.23 (0.68)	< 0.001
	Effect size^b^	0.012	0.008	0.008	0.006	
Supervisor support	Elementary school	8.46 (2.21)	8.48 (2.22)	8.49 (2.24)	8.49 (2.25)	< 0.001
	Junior high school	8.32 (2.28)	8.34 (2.29)	8.31 (2.31)	8.28 (2.33)	< 0.001
	High school	7.78 (2.27)	7.83 (2.30)	7.84 (2.32)	7.89 (2.34)	< 0.001
	Special needs school	7.66 (2.21)	7.72 (2.23)	7.72 (2.24)	7.74 (2.25)	0.006
	Effect size^b^	0.018	0.016	0.016	0.014	
Co-worker support	Elementary school	9.11 (2.02)	9.10 (2.04)	9.10 (2.05)	9.08 (2.07)	0.004
	Junior high school	8.79 (2.10)	8.81 (2.12)	8.76 (2.14)	8.72 (2.16)	< 0.001
	High school	8.44 (2.09)	8.49 (2.12)	8.48 (2.14)	8.45 (2.16)	0.006
	Special needs school	8.54 (2.03)	8.56 (2.07)	8.55 (2.08)	8.51 (2.09)	0.194
	Effect size^b^	0.017	0.014	0.015	0.015	
Support from family and friends	Elementary school	10.14 (1.99)	10.18 (2.00)	10.18 (2.01)	10.18 (2.02)	< 0.001
	Junior high school	9.82 (2.17)	9.86 (2.18)	9.85 (2.20)	9.83 (2.21)	0.045
	High school	9.65 (2.22)	9.69 (2.24)	9.68 (2.27)	9.67 (2.29)	0.167
	Special needs school	9.66 (2.22)	9.71 (2.20)	9.72 (2.21)	9.72 (2.22)	0.027
	Effect size^b^	0.010	0.010	0.010	0.010	
Stress responses	Elementary school	55.98 (14.6)	55.25 (14.6)	56.00 (15.0)	56.70 (15.3)	< 0.001
	Junior high school	57.39 (15.4)	56.32 (15.3)	57.19 (15.8)	58.13 (16.1)	< 0.001
	High school	56.28 (15.4)	55.63 (15.5)	56.10 (15.8)	56.58 (16.1)	< 0.001
	Special needs school	55.87 (15.1)	55.39 (15.2)	56.02 (15.5)	56.31 (15.6)	< 0.001
	Effect size^b^	0.002	0.001	0.001	0.002	

BJSQ, Brief Job Stress Questionnaires; M, mean; SD, standard deviation.

a Welch's one-way ANOVA was performed to examine the difference of scores between the yeas for each school type. Effect sizes [eta-squared value (η^2^) was calculated as the effect size for one-way ANOVA] were negligible (η^2^ < 0.01) for all scales.

b Welch's one-way ANOVA was performed to examine the difference of scores between different school types for each year. P-values were less than 0.001 for all scales. Eta-squared value was calculated as the effect size for one-way ANOVA. Higher scores indicate higher stress levels for the quantitative and qualitative workloads, interpersonal conflict (scores range between 3.0 and 12.0, respectively), physical demands, and poor physical environment (scores range between 1.0 and 4.0, respectively). Higher scores indicate better work situation for job control (scores range between 3.0 and 12.0), skill utilization, suitable jobs, and meaningfulness of work (scores range between 1.0 and 4.0, respectively). Regarding buffering factors, higher scores indicate higher levels of social support (scores range between 3.0 and 12.0).

The stress response scores of junior high school teachers were the highest, followed by those of elementary school teachers. Special needs school teachers' scores were the lowest among all school types. However, the difference in stress response scores between school types was negligibly small in all four years (η^2^ = 0.001–0.002).

Welch's ANOVA showed a significant difference in almost all the scales between 2019, 2020, 2021, and 2022 (*p* < 0.05); nevertheless, the effect size of the difference between years was marginally small for all the scales (η^2^ < 0.01). The scores for workloads (quantitative and qualitative) and stress response decreased from 2019 to 2020 and increased from 2020 to 2022 in a consistent pattern in all school types although the changes in scores were minimal.

### 3.5. Relationship between the BJSQ job stress scales and stress response scores among public school teachers by each school type in the third year of the pandemic (2022)

Table 3 displays the results of the multiple regression analysis assessing the association between the BJSQ job stress scales and stress response scores after adjusting for the effects of gender and years of experience as a teacher. First, we examined the correlation coefficients for each pair of predictor variables, none of which was 0.8 or above. In addition, all VIFs were below 5.0; therefore, multicollinearity was ruled out.

**Table 3:** Multiple regression analysis which examined the relationship between the BJSQ job stress scales and stress response scores among public school teachers in the third year of the pandemic (2022), adjusting for gender and years of experience as a teacher.

**Scales**	**Elementary school**	**Junior high school**	**High school**	**Special needs school**	**Significantly different βs^a^**
Years of experience	−0.028	−0.037	−0.038	−0.008^†^	E–J, E–H, E–S, J–S, H–S
Gender (reference: Men)	0.051	0.075	0.076	0.059	E–J, E–H, J–S, H–S
Quantitative workload	0.187	0.178	0.185	0.193	
Qualitative workload	0.146	0.155	0.151	0.129	E–J, E–S, J–S, H–S
Physical demands	0.058	0.054	0.039	0.053	E–H, J–H, H–S
Interpersonal conflict	0.141	0.145	0.139	0.172	E–S, J–S, H–S
Poor physical environment	0.076	0.075	0.073	0.065	E–S, J–S
Job control	−0.133	−0.145	−0.142	−0.125	E–J, J–S
Suitable jobs	−0.021	0.017	−0.016	−0.020	
Skill utilization	−0.139	−0.132	−0.124	−0.118	E–H, E–S
Meaningfulness of work	−0.116	−0.115	−0.127	−0.105	H–S
Supervisor support	−0.022	−0.020	−0.031	−0.029	
Co-worker support	−0.037	−0.046	−0.038	−0.048	
Support from family and friends	−0.096	−0.097	−0.086	−0.104	E–H, J–H, H–S
*R* ^2^	0.445	0.485	0.500	0.461	

BJSQ, Brief Job Stress Questionnaires; E, Elementary school; J, Junior high school; H, High school; S, Special needs school; R^2^, Adjusted R square. Standardized regression coefficient (β) is shown in each category. All regression coefficients were statistically significant (*p* < 0.001) except years of experience among special needs school teachers.

† β was not statistically significant (*p* = 0.125).

a The difference in regression coefficients between school types was statistically significant [e.g., “E–J” means the difference in regression coefficients between elementary (E) and junior high school (J) was statistically significant (*p* < 0.05)].

All regression coefficients were statistically significant (*p* < 0.001) except for years of experience among teachers in special needs schools. Gender (being a woman) was positively associated with stress response scores in all school types. The association between gender and stress response scores was significantly stronger among junior high and high school teachers (β = 0.075–0.076) than those among elementary and special needs school teachers (β = 0.051–0.059).

Quantitative workload was the most significant positive predictor of stress responses among schoolteachers regardless of school type (β = 0.178–0.193), followed by qualitative workload (β = 0.129–0.155). In special needs schools, interpersonal conflict among teachers was the second salient factor leading to stress responses (β = 0.172). The association between interpersonal conflict and stress responses among teachers in special needs schools was significantly stronger than among teachers in other school types. Job control was the most buffering factor for teachers' stress responses in all school types (β = −0.145–−0.125).

### 3.6. Public school teachers' perceived main stressors by school types in the third year of the pandemic (2022)

Table 4 exhibits public school teachers' primary sources of stress in 2022 by school type. The highest percentage of teachers indicated a “workload of clerical tasks” as their main stressor regardless of school type (18.7–21.4%). The association between teachers' main stressor categories and school types was practically significant in “dealing with difficult students,” “dealing with challenging parents,” and “extra-curricular club activity” (Cramer's V = 0.103–0.305).

**Table 4:** Public school teachers' main stressors in the third year of the pandemic (2022) by school types.

**Main stressor**		**Elementary school (*N* = 110,305)**	**Junior high school (*N* = 62,459)**	**High school (*N* = 40,843)**	**Special needs school (*N* = 18,970)**	**Cramer's V**
Dealing with difficult students	Count	29,015	11,639	5,672	2,418	
	% (within the school)	26.3%	18.6%	13.9%	12.7%	**0.133**
	Adjusted residual	60.2	−16.7	−38.7	−29.0	
Workload of clerical tasks	Count	22,635	13,295	8,726	3,544	
	% (within the school)	20.5%	21.3%	21.4%	18.7%	0.018
	Adjusted residual	−2.3	4.0	3.5	−7.2	
Dealing with challenging parents	Count	16,300	7,670	2,450	1,538	
	% (within the school)	14.8%	12.3%	6.0%	8.1%	**0.103**
	Adjusted residual	38.8	2.3	−41.2	−17.3	
School management duties	Count	16,074	9,859	7,380	3,295	
	% (within the school)	14.6%	15.8%	18.1%	17.4%	0.037
	Adjusted residual	−14.7	0.4	14.2	6.4	
Responsibility for students' learning	Count	12,024	4,688	4,487	1,757	
	% (within the school)	10.9%	7.5%	11.0%	9.3%	0.050
	Adjusted residual	15.8	−23.2	8.3	−2.9	
Extra-curricular club activities	Count	968	12,076	5,744	106	
	% (within the school)	0.9%	19.3%	14.1%	0.6%	**0.305**
	Adjusted residual	−121.5	119.9	48.4	−39.8	
Demonstration lessons	Count	8,909	3,570	1,034	964	
	% (within the school)	8.1%	5.7%	2.5%	5.1%	0.085
	Adjusted residual	35.1	−6.2	−34.0	−6.8	
Relationship with co-workers	Count	10,080	6,667	4,373	3,419	
	% (within the school)	9.1%	10.7%	10.7%	18.0%	0.076
	Adjusted residual	−21.1	1.2	1.1	35.0	
Relationship with supervisors	Count	6,102	3,800	1,707	1,049	
	% (within the school)	5.5%	6.1%	4.2%	5.5%	0.028
	Adjusted residual	1.8	8.3	−12.4	0.6	
Unfamiliar work environment	Count	5,832	3,248	2,038	1,269	
	% (within the school)	5.3%	5.2%	5.0%	6.7%	0.019
	Adjusted residual	−0.8	−1.6	−3.3	8.7	
Long commuting time	Count	4,037	2,847	2,888	1,145	
	% (within the school)	3.7%	4.6%	7.1%	6.0%	0.061
	Adjusted residual	−22.4	−1.9	25.0	9.1	
Personal problems	Count	11,186	5,384	4,016	2,418	
	% (within the school)	10.1%	8.6%	9.8%	12.7%	0.036
	Adjusted residual	3.8	−12.4	−0.4	13.7	

The number of cases is shown with their percentage in each category. A chi-squared test showed that the association between main stressors and school types was statistically significant in all stressor categories (*p* < 0.001). Values in bold indicate the absolute value of Cramer's V is more than 0.1.

The percentage of elementary school teachers who indicated “dealing with difficult students” as their main stressor was the highest (26.3%) among all school types, followed by junior high school teachers (18.6%). Similarly, the percentage of elementary school teachers who indicated “dealing with challenging parents” as their main stressor was the highest (14.8%), followed by junior high school teachers (12.3%). Meanwhile, the percentage of junior high school teachers who perceived “extra-curricular club activities” as their main stressor was the highest (19.3%), followed by high school teachers (14.1%). The highest percentage of special needs school teachers indicated “relationship with co-workers” as their main stressor (18.0%) even though the effect size of its association with school types was negligible (Cramer's V = 0.076).

## 4. Discussion

This study aimed to assess public school teachers' occupational stress and clarify stress factors considering the differences in school types during the prolonged pandemic period. The results revealed that, regardless of school type, quantitative workload was the most significant factor for teachers' stress responses. Moreover, the results unveiled significant differences in the impact of each stress factor on teachers' stress responses between school types. To the best of our knowledge, this is the first study to investigate schoolteachers' work-related stress by school type using a large-scale nationwide survey data with an adequately high participation rate of the target population in Japan.

The results indicated that stress response scores among teachers increased significantly as working hours increased regardless of school type. In addition, multiple regression analysis demonstrated that quantitative workload was the most significant positive predictor of stress responses among teachers in all school types, thus supporting Hypothesis 1. These findings are consistent with those of previous studies (15, 52); quantitative workload and long working hours are significantly associated with psychological stress reactions among schoolteachers (15, 16).

The association between teachers' work overload and their mental health problems have been indicated worldwide (9, 53). A study in German revealed that teachers who worked more than 45 h per week suffered more often from unrecoverable fatigue than teachers who worked <40 h per week (53). A study in Philippine demonstrated that excessive workload among schoolteachers significantly increased their burnout rate (9). According to the Teacher Workload Survey 2019 (conducted in England), approximately 70% of primary school teachers and 90% of secondary school teachers reported that their workload was a serious problem (54). Thus, the present study further highlighted the importance of addressing teachers' excessive workload, which has been a serious social concern globally, for safeguarding teachers' mental health.

Previous studies have shown that teachers are burdened with a substantial amount of administrative and clerical tasks in addition to teaching duties (17, 18). Even in this study, the highest percentage of teachers indicated a “workload of clerical tasks” and “school management duties” as a main stressor regardless of school type. Paperwork related to educational and other peripheral tasks are perceived as considerably stressful for schoolteachers globally (54, 55). A survey in England demonstrated that most primary and secondary school teachers recognized their spending “too much” time on administrative work and related clerical tasks (54). Moreover, teaching-related paperwork significantly contributed to schoolteachers' occupational stress (55). This situation is particularly true for the school workplace in Japan. The time spent on clerical and other related tasks was approximately 5.6 h work per week among teachers in Japan, more than double the average for all investigated countries (18). Our previous study demonstrated that teachers working overtime to conduct core educational work and peripheral tasks (e.g., clerical tasks) exhibited significantly higher stress responses than those engaging only in core educational work (52). To reduce teachers' occupational stress, more support staff members who can help with teachers' peripheral tasks must be employed. In addition, policymakers should re-examine the necessity of paperwork duties imposed on teachers and take effective measures to reduce this burden.

We hypothesized that junior high school teachers would experience the highest levels of stress, is similar to the special needs of school teachers. However, the results did not demonstrate a significant difference in schoolteachers' stress levels between different school types despite those in junior high schools having the highest levels. Therefore, Hypothesis 2 is not supported.

As expected, the working hours of junior high school teachers were the longest, followed by those of elementary school teachers, consistent with previous studies (18, 32). Furthermore, the highest percentage of junior high school teachers perceived “extra-curricular club activities” as their main stressor. Globally, extra-curricular club activities are considered as an integral component of school life, especially for secondary school students (56, 57). Extra-curricular activities have positive impacts on students' academic performance, regular class attendance, and favorable self-image among peers (56). Meanwhile, these activities place considerable burden on teachers involved (57). Extra-curricular club activities are conducted fervently in Japanese junior high schools, and many teachers serve in these activities as supervisors (31). Average hours spent on engaging in extra-curricular activities are extremely long (7.6 h per week) among junior high school teachers (elementary school teachers spend only 0.6 h per week) (32). According to international organizations such as the OECD, one of the strengths of Japan's public school system is that it provides students with holistic educational opportunities through various extra-curricular activities, including school trips, clubs, and school festivals (58). However, this situation imposes a substantial burden on public school teachers in Japan (58). To reduce teachers' excessive workloads, MEXT instructed local governments to gradually transfer the administration of weekend club activities to private sports clubs in local communities over several years starting from 2023 (59). However, there are numerous issues that must be addressed in transferring these club activities to local communities. The availability of personnel and facilities for managing these activities is inadequate depending on the region. In addition, outsourcing school activities to private clubs imposes new expenses on parents (59). The financial problems associated with extra-curricular activities, especially for low-income households, have also been identified in other countries (60, 61). Therefore, the government and policymakers should secure a sufficient budget to support the collaborating private clubs and low-income households to establish a sustainable model for these club activities.

The results revealed that quantitative and qualitative workloads of elementary school teachers were the largest among all school types. Moreover, the highest percentage of elementary school teachers perceived “dealing with difficult students” as their main stressor. In the Japanese educational system, elementary school teachers generally teach almost all subjects, from math and science to art class, while engaging in various extra duties. These duties include attending to students who are absent from school, providing guidance regarding their daily lives, and communicating with parents or guardians (52). Furthermore, the Japanese government has established an inclusive education system (one that encourages students with and without disabilities to learn together as much as possible) to meet the requirements of Article 24 of the Convention on the Rights of Persons with Disabilities. Based on these requirements, an increasing number of students with special needs are enrolled in regular public schools (62). This trend is particularly noticeable in elementary schools in Japan. According to a government report, the percentage of students with learning difficulties or behavioral problems taught in regular class settings was significantly higher in elementary schools (10.4%) than in secondary schools (5.6% in junior high schools and 2.2% in high schools) (63). Addressing students with special needs is creating additional challenges for primary school teachers already overloaded with various school duties. Schoolteachers' stress related to the implementation of inclusive education has also been reported in surveys from other nations (64, 65). A study in Ireland revealed that more than 80% of primary school teachers perceived educating children with behavior difficulties as increasingly challenging and stressful (64).

Despite challenging working conditions, the class size in Japanese schools remains relatively large, and the student-teacher ratio is considerably high in Japan compared with other OECD participating countries (66). The lack of an adequate number of schoolteachers compared to the number of students negatively affects the quality of education and teachers' work-related stress. Considering these conditions, increasing the number of schoolteachers and support staff is crucial to safeguard teachers' mental health. In addition, given the global trend of accepting students' individual needs, providing all teachers with opportunities to acquire basic special needs education skills and sufficient mental support is imperative.

The results demonstrated that the scores for interpersonal conflict among teachers were the highest among special needs schools. In addition, multiple regression analysis showed that interpersonal conflict was the second most important factor leading to stress responses among teachers in special needs schools. Furthermore, the percentage of teachers who perceived “relationship with co-workers” as their main stressor was the highest among special needs schools. Therefore, Hypothesis 3 is fully supported.

Conflicts among co-workers have been linked to teachers' burnout, which is directly associated with teachers going on sick leave due to mental disorders (12, 13). Team teaching is an instructional strategy in which two or more teachers collaborate to teach the same group of students (67). This strategy is commonly employed in many special needs schools in Japan. If effectively used, collaborative exchanges between teachers can enhance their professional work and reduce their workload (68). Moreover, students instructed through collaborative teaching achieve higher academic outcomes and support from teaching staff (68). Considering its promising potential, team teaching has received increased attention globally (67). Meanwhile, teachers' stress levels may increase if teachers with different teaching style preferences are forced to collaborate (34). Muehlbacher et al. demonstrated that team teaching is an educational practice requiring teachers to have increased emotion regulation (69). Team teachers frequently use emotion regulation techniques, such as attentional deployment and reappraisal, to minimize experiencing negative emotions. Additionally, a positive discussion with a partner teacher after class is regularly used to address disagreements among team teachers (69). Taniguchi et al. demonstrated that a postponed-solution coping strategy reduced schoolteachers' stress caused by interpersonal problems with co-workers (70). Assertiveness, a social communication skill that openly expresses oneself while being concerned with others, can increase teachers' wellbeing at work (71). In this context, a stress coping program that focuses on relationship problems among colleagues would be significantly useful in reducing schoolteachers' work-related stress, especially for teachers involved in team teaching. Therefore, acquiring effective communication skills between colleagues is crucial for managing teachers' occupational stress.

In this study, the scores of teachers' workloads (quantitative and qualitative) and stress responses were the highest in 2022 (the third year of the pandemic) in all school types, although the effect size of the difference between the years was marginal. Moreover, in the same working hours category, the stress response scores in 2022 were the highest, regardless of school type. The results indicated that schoolteachers experienced significant work-related stress during the prolonged pandemic. Therefore, Hypothesis 4 is partially supported.

The scores for teachers' stress responses and workloads temporarily dropped in 2020 (the first year of the pandemic) in all school types, possibly due to the cancelation of various school events or activities and a decrease in schoolteacher related tasks (72). However, many school events and activities that were canceled in 2020 were reinstated in 2021 at Japanese public schools. In 2021 and 2022, COVID-19 variants continued to spread throughout Japan. Infection control measures, such as social distancing, were implemented to prevent infection, while many school activities and events were reinstituted (72). Additionally, online teaching has been implemented in place of traditional in-person learning since the COVID-19 outbreak (73). Teachers experienced elevated stress levels as a result of the unfamiliar workload entailed by online education methods (74). These difficult situations may have contributed to an increase in schoolteachers' stress levels during the prolonged pandemic. Furthermore, a study in China revealed that schoolteachers remain under considerable pressure even after the end of COVID-19 restrictions (75). Many offline school activities were arranged in the short period of time after the restrictions were lifted, imposing a substantial workload on teachers (75). The pandemic also caused severe psychological trauma among schoolteachers (75). Considering the possible prolonged impacts of the pandemic on teachers' mental health, their stress levels must be monitored throughout and after the pandemic.

The COVID-19 pandemic has severely affected the global economy (76), including the Japanese economy (77). However, public spending on primary, secondary, and tertiary education in 2019 was 7.8% of the total government expenditure in Japan, which was relatively low compared with other OECD countries (the OECD average was 10.6%) (66). Thus, the government should supplement public spending on school resources, such as by increasing the number of schoolteachers and support staff, and by providing teachers with opportunities to learn effective stress coping skills.

Although this study offers several important insights, it has some limitations. This study comprises four cross-sectional studies, executing a comparative analysis between years based on these survey data. The dataset consisted of repeated cross-sectional data that precluded the examination of individual-level changes prior to and after the onset of the pandemic. Longitudinal studies based on solid panel data obtained before and during the pandemic are required to identify the effects of the pandemic on teachers' occupational stress accurately. Nonetheless, considering the high participation rate in the “Stress Check” survey, it is plausible that a significant number of public school teachers completed the surveys in all four years. This study investigated occupational stress among public school teachers, including elementary, junior high, high, and special needs school teachers. The results may differ in other school settings, such as private schools, colleges, and universities. The pandemic-related stress may have differed among schoolteachers with administrative positions and clerical staff. Stress structures among schoolteachers may also differ in other cultures and countries. Planning cohort studies investigating cross-cultural differences in teachers' occupational stress should be valuable for this field of research. Further well-designed studies including these variables are necessary to counteract these possible biases. Despite these limitations, we believe that this study will provide useful proposals in this field of research.

## 5. Conclusion

The present study investigated public school teachers' work-related stress, considering the differences in school types. Regardless of school setting, quantitative workload and long work hours were the most significant factors for teachers' stress responses. This study further highlighted the importance of reducing teachers' workload for addressing their occupational stress. Meanwhile, stress factors among teachers significantly varied between school types. The highest percentage of junior school teachers perceived “extra-curricular club activities” as their main stressor. The scores for teachers' job workload were the highest in elementary schools, and the highest percentage of elementary school teachers perceived “dealing with difficult students” as their main stressor. Moreover, teachers' interpersonal conflict scores were higher in special needs schools than in any other school type. Considering the global attention on team teaching in educational institutions, acquiring effective communication skills between colleagues is crucial for managing teachers' occupational stress. Finally, teachers' workload and stress levels significantly increased in the third year of the pandemic (2022) compared to the pre-pandemic year (2019), although the difference were minimal. Given the possible prolonged impacts of the pandemic on teachers' stress, teachers' stress levels must be monitored throughout and after the pandemic. These findings suggest that increasing the number of schoolteachers and support staff as well as providing adequate organizational support are critical to prevent teachers' sick leave due to mental disorders. Additionally, taking comprehensive countermeasures against teachers' occupational stress, considering the differences in school types, is crucial for safeguarding teachers' mental health.

## Data availability statement

The datasets presented in this article are not readily available because we cannot publicly present individual data due to a data provider's regulations. Qualifying researchers may apply to access a minimal dataset on reasonable request by contacting the corresponding author. Requests to access the datasets should be directed to KT, tubonok@tokaihp.jp.

## Ethics statement

The studies involving humans were approved by the Institutional Review Board of Tokai Central Hospital. The studies were conducted in accordance with the local legislation and institutional requirements. The Ethics Committee/Institutional Review Board waived the requirement of written informed consent for participation from the participants or the participants' legal guardians/next of kin because This study used existing data for the study, and these data were already completely anonymized and untraceable.

## Author contributions

KT: Conceptualization, Data curation, Formal analysis, Funding acquisition, Investigation, Methodology, Project administration, Supervision, Writing—original draft, Writing—review and editing. SM: Supervision, Writing—review and editing.

## References

[1] Titheradge D Hayes R Longdon B Allen K Price A Hansford L. Psychological distress among primary school teachers: a comparison with clinical and population samples. Public Health. (2019) 166:53–6. 10.1016/j.puhe.2018.09.022 30448692

[2] Embse N Ryan SV Gibbs T Mankin A. Teacher stress interventions: a systematic review. Psychol Sch. (2019) 56:1328–43. 10.1002/pits.22279

[3] Stansfeld SA Rasul FR Head J Singleton N. Occupation and mental health in a national UK survey. Soc Psychiatry Psychiatr Epidemiol. (2011) 46:101–10. 10.1007/s00127-009-0173-7 20033130 PMC3034883

[4] Dalia D Heba A. Occupational stress, anxiety and depression among Egyptian teachers. J Epidemiol Glob Health. (2017) 7:191–8. 10.1016/j.jegh.2017.06.002 28756829 PMC7320446

[5] Jendle H Wallnäs A. Effects of Exercise, Social Support and Hardiness on Occupational Stress in Swedish Teachers (Bachelor Thesis). Örebro: Örebro University (2017).

[6] Steiner ED Woo A. Job-Related Stress Threatens the Teacher Supply: Key Findings from the 2021 State of the U.S. Teacher Survey. Santa Monica, CA: RAND Corporation (2021).

[7] Maslach C Leiter MP. Understanding the burnout experience: recent research and its implications for psychiatry. World Psychiatry. (2016) 15:103–11. 10.1002/wps.20311 27265691 PMC4911781

[8] García-Carmona M Marín MD Aguayo R. Burnout syndrome in secondary school teachers: a systematic review and meta-analysis. Soc Psychol Educ. (2019) 22:189–208. 10.1007/s11218-018-9471-9

[9] Jomuad PD Antiquina MM Cericos EU Bacus JA Vallejo JH Dionio BB. Teachers' workload in relation to burnout and work performance. Int J Edu Pol Res Rev. (2021) 8:48–53. 10.15739/IJEPRR.21.007

[10] Leung SS Chiang VC Chui YY Mak YW Wong DF A. brief cognitive-behavioral stress management program for secondary school teachers. J Occup Health. (2011) 53:23–35. 10.1539/joh.L10037 21079374

[11] Aldrup K Klusmann U Lüdtke O Göllner R Trautwein U. Student misbehavior and teacher well-being: testing the mediating role of the teacher-student relationship. Learn Instr. (2018) 58:126–36. 10.1016/j.learninstruc.2018.05.006

[12] Sulea C Filipescu A Horga O Fischmann G. Interpersonal mistreatment at work and burnout among teachers. Cogn Brain Behav Interdiscip J. (2012) 16:553–70.

[13] Bruce P Bruce C Hrymak V Hickey N Mannix McNamara P. Staff stress and interpersonal conflict in secondary schools—implications for school leadership. Societies. (2022) 12:186. 10.3390/soc12060186

[14] Allen R Benhenda A Jerrim J Sims S. New evidence on teachers' working hours in England. An empirical analysis of four datasets. Res Pap Educ. (2021) 36:657–81. 10.1080/02671522.2020.1736616

[15] Matsushita M Yamamura S. The relationship between long working hours and stress responses in junior high school teachers: a nationwide survey in Japan. Front Psychol. (2022) 12:775522. 10.3389/fpsyg.2021.775522 35087451 PMC8786715

[16] Bannai A Ukawa S Tamakoshi A. Long working hours and psychological distress among school teachers in Japan. J Occup Health. (2015) 57:20–7. 10.1539/joh.14-0127-OA 25422128

[17] Ibrahim RZAR Zalam WZM Dagang MM Omar K Bakar AA Ali SNM. Predicting psychological distress: a cross-sectional study of Malaysian teachers. Eur J Mol Clin Med. (2020) 6:505–15.

[18] Organization for Economic Co-operation and Development. TALIS 2018 Results Volume I. Teachers and School Leaders as Lifelong Learners. Paris: OECD Publishing (2019).

[19] Timms C Graham D Caltabiano M. Gender implication of perceptions of trustworthiness of school administration and teacher burnout/job stress. Aust J Soc Issues. (2006) 41:343–58. 10.1002/j.1839-4655.2006.tb00020.x

[20] Chan A Chen K Chong E. Work stress of teachers from primary and secondary schools in Hong Kong. Lect Notes Comput Sci. (2010) 15:2182. 20331916 10.1080/10803548.2010.11076825

[21] Besse R Howard K Gonzalez S Howard J. Major depressive disorder and public school teachers: evaluating occupational and health predictors and outcomes. J Appl Biobehav Res. (2015) 20:71–83. 10.1111/jabr.12043

[22] Calsamiglia C Loviglio A. Maturity and school outcomes in an inflexible system: evidence from Catalonia. SERIEs. (2020) 11:1–49. 10.1007/s13209-019-0196-6 32226557 PMC7096368

[23] Agyapong B Obuobi-Donkor G Burback L Wei Y. Stress, burnout, anxiety and depression among teachers: a scoping review. Int J Environ Res Public Health. (2022) 19:10706. 10.3390/ijerph191710706 36078422 PMC9518388

[24] Kavita K Hassan N. Work stress among teachers: a comparison between primary and secondary school teachers. Int J Acad Res Progress Educ Dev. (2018) 7:60–6. 10.6007/IJARPED/v7-i4/4802

[25] Kongcharoen J Onmek N Jandang P Wangyisen S. Stress and work motivation of primary and secondary school teachers. J Appl Res High Educ. (2019) 12:709–23. 10.1108/JARHE-04-2019-0088

[26] Strydom L Nortje N Beukes R Esterhuyse K Westhuizen J. Job satisfaction amongst teachers at special needs schools. S Afr J Educ. (2011) 32:255–66. 10.15700/saje.v32n3a582

[27] Crispel O Kasperski R. The impact of teacher training in special education on the implementation of inclusion in mainstream classrooms. Int J Incl Educ. (2021) 25:1079–90. 10.1080/13603116.2019.1600590

[28] Kristiana I Hendriani W. Teaching efficacy in inclusive education (IE) in Indonesia and other Asia, developing countries: a systematic review. J Educ Learn. (2018) 12:166. 10.11591/edulearn.v12i2.7150

[29] Ministry of Education Culture Sports Science Technology. Personnel Administration Status Survey of Public School Staff. (2021). Available online at: https://www.mext.go.jp/a_menu/shotou/jinji/1411820_00005.htm (accessed May 10, 2023).

[30] Yamanaka S Suzuki KH. Japanese Education Reform Towards Twenty-First Century Education. In:Reimers FM, editor. Audacious Education Purposes: How Governments Transform the Goals of Education Systems. Cham: Springer International Publishing (2020). p.81–103.

[31] Hojo M. Association between student-teacher ratio and teachers' working hours and workload stress: evidence from a nationwide survey in Japan. BMC Public Health. (2021) 21:1635. 10.1186/s12889-021-11677-w 34493251 PMC8422828

[32] Ministry of Education Culture Sports Science Technology. Report on the Survey on the Working Conditions of Public School Teachers. (2023). Available online at: https://www.mext.go.jp/content/20230428-mxt_zaimu01-100003067-2.pdf (accessed July 20, 2023).

[33] Takahashi M. Sociomedical problems of overwork-related deaths and disorders in Japan. J Occup Health. (2019) 61:269–77. 10.1002/1348-9585.12016 30977205 PMC6620752

[34] Ogawa S Kawamura R Kojima M. Stress and resilience of Japanese teachers in special needs schools for students with intellectual disabilities during the COVID-19 pandemic. Front Educ. (2022) 7:869876. 10.3389/feduc.2022.869876

[35] Kawakami N Tsutsumi A. The stress check program: a new national policy for monitoring and screening psychosocial stress in the workplace in Japan. J Occup Health. (2016) 58:1–6. 10.1539/joh.15-0001-ER 26607455

[36] Ozamiz-Etxebarria N Berasategi Santxo N Idoiaga Mondragon N Dosil SM. The psychological state of teachers during the COVID-19 crisis: the challenge of returning to face-to-face teaching. Front psychol. (2021) 11:620718. 10.3389/fpsyg.2020.620718 33510694 PMC7835279

[37] Silva DFO Cobucci RN Lima SCVC de Andrade FB. Prevalence of anxiety, depression, and stress among teachers during the COVID-19 pandemic: a PRISMA-compliant systematic review. Medicine. (2021) 100:27684. 10.1097/MD.0000000000027684 PMC8568426 34871251

[38] Kitahara K Nishikawa Y Yokoyama H Kikuchi Y Sakoi M. An overview of the reclassification of COVID-19 of the infectious diseases control law in Japan. Glob Health Med. (2023) 5:70–4. 10.35772/ghm.2023.01023 37128229 PMC10130540

[39] Japan Industrial Safety Health Association. The Brief Job Stress Questionnaire. N/A. (2022). Available online at: https://www.jisha.or.jp/english/topics/202108_16.html (accessed June 23, 2022).

[40] Hirokawa K Ohira T Kajiura M Imano H Kitamura A Kiyama M. Job stress factors measured by Brief Job Stress Questionnaire and sickness absence among Japanese workers: a longitudinal study. Fukushima J Med Sci. (2020) 66:88–96. 10.5387/fms.2019-15 32595178 PMC7470755

[41] Inoue A Kawakami N Shimomitsu T Tsutsumi A Haratani T Yoshikawa T. Development of a short questionnaire to measure an extended set of job demands, job resources, and positive health outcomes: the new brief job stress questionnaire. Ind Health. (2014) 52:175–89. 10.2486/indhealth.2013-0185 24492763 PMC4209588

[42] Hurrell JJ McLaney MA. Exposure to job stress: a new psychometric instrument. Scand J Work Environ Health. (1988) 14:27–8. 3393871

[43] Doef M Verhoeven C. The Job Demand-Control (-Support) Model in the Teaching Context. In:McIntyre T McIntyre S Francis D, editor. Educator Stress. Aligning Perspectives on Health, Safety and Well-Being. Cham: Springer International Publishing (2017), p.197–222.

[44] Shimomitsu T. The Final Development of the Brief Job Stress Questionnaire Mainly Used for Assessment of the Individuals. Ministry of Labour Sponsored Grant for The Prevention of Work-Related Illness: The 1999 Report. Tokyo: Tokyo Medical College (2000), p.126–64.

[45] Wada K Sairenchi T Haruyama Y Taneichi H Ishikawa Y Muto T. Relationship between the onset of depression and stress response measured by the brief job stress questionnaire among Japanese employees: a cohort study. PLoS ONE. (2013) 8:56319. 10.1371/journal.pone.0056319 23424656 PMC3570457

[46] Kawano Y. Association of job-related stress factors with psychological and somatic symptoms among Japanese hospital nurses: effects of departmental environment in acute care hospitals. J Occup Health. (2008) 50:79–85. 10.1539/joh.50.79 18285650

[47] Umehara K Ohya Y Kawakami N Tsutsumi A Fujimura M. Association of work-related factors with psychosocial job stressors and psychosomatic symptoms among Japanese pediatricians. J Occup Health. (2007) 49:467–81. 10.1539/joh.49.467 18075207

[48] Muto S Muto T Seo A Yoshida T Taoda K Watanabe M. Job stressors and job stress among teachers engaged in nursing activity. Ind Health. (2007) 45:44–8. 10.2486/indhealth.45.44 17284873

[49] Mitani S. Comparative analysis of the Japanese version of the revised impact of event scale: a study of firefighters. Prehosp Disaster Med. (2008) 23:s20–6. 10.1017/S1049023X00024055 18702284

[50] Cohen J. Statistical Power Analysis for the Behavioral Sciences. New York, NY: Lawrence Erlbaum Associates. (1988).

[51] Xαρíτoυ A Haritou A Clogg C Petkova E. Statistical methods for comparing regression coefficients between models. Am J Sociol. (1995) 100:1261–93. 10.1086/230638

[52] Furihata R Kuwabara M Oba K Watanabe K Takano N Nagamine N. Association between working overtime and psychological stress reactions in elementary and junior high school teachers in Japan: a large-scale cross-sectional study. Ind Health. (2022) 60:133–45. 10.2486/indhealth.2021-0069 34645742 PMC8980699

[53] Kreuzfeld S Felsing C Seibt R. Teachers' working time as a risk factor for their mental health - findings from a cross-sectional study at German upper-level secondary schools. BMC Public Health. (2022) 22:307. 10.1186/s12889-022-12680-5 35164735 PMC8845294

[54] Walker M Worth J Brande JV. Teacher Workload Survey 2019: Research Report. (2019). Available oline at: https://www.gov.uk/government/publications/teacher-workload-survey-2019 (accessed August 10, 2023).

[55] Merasul JH Cathy Mae DT. Teaching-related paperwork: examining linkage to occupational stress of public school teachers in primary education. ASEAN J Basic High Educ. (2021) 5:13–5.

[56] Anjum S. Impact of extracurricular activities on academic performance of students at secondary level. Int J Appl Guid Couns. (2021) 2:7–14. 10.26486/ijagc.v2i2.1869

[57] Learning S. Teacher Workloads Leave Students Missing Out on Extra-Curricular Activities. (2020). Available online at: https://sparx-learning.com/teacher-workloads-leave-students-missing-out-on-extra-curricular-activities/ (accessed September 10, 2023).

[58] Masatoshi S. Japanese Teachers at the Breaking Point: Long Hours Blamed for Growing Shortage. (2023). Available online at: https://www.nippon.com/en/in-depth/d00887/ (accessed June 20, 2023).

[59] Kobayashi Y. Japan Looks to Shift Operation of Public Junior High Club Activities to Local Communities. (2022). Available online at: https://mainichi.jp/english/articles/20220606/p2a/00m/0na/002000c (accessed June 20, 2023).

[60] Hjalmarsson S. Pay to play? Economic constraints and participation in extracurricular activities. Eur Sociol Rev. (2022) 39:586–600. 10.1093/esr/jcac061

[61] Kim J. An Analysis of extra-curricular activities in childcare facilities and the factors affecting on expenses of extra-curricular activities. J Korean Child Care Educ. (2014) 10:5–23. 10.14698/jkcce.2014.10.5.005

[62] Furuta H Osugi N. Developing an inclusive education system in Japan : the case of Yamaga City, Kumamoto. Bull Fac Educ Kumamoto Univ. (2016) 65:139–44.

[63] Ministry of Education Culture Sports Science Technology. The Survey on Students With Special Needs Who are Instructed in Reglular Classes. (2022). Available online at: https://www.mext.go.jp/b_menu/houdou/2022/1421569_00005.htm (accessed July 20, 2023).

[64] Morgan M Craith DN. Workload, stress and resilience of primary teachers: report of a survey of INTO members. Irish Teachers J. (2015) 1:9–20.

[65] Candeias AA Galindo E Calisto I Borralho L Reschke K. Stress and burnout in teaching. Study in an inclusive school workplace. Health Psychol Rep. (2021) 9:63–75. 10.5114/hpr.2020.100786 PMC10694697 38084117

[66] *Organization for Economic Co-operation and Development*. Education at a Glance 2021: OECD indicators. Paris: OECD Publishing (2021).

[67] Decuyper A Tack H Vanblaere B Simons M Vanderlinde R. Collaboration and shared responsibility in team teaching: a large-scale survey study. Educ Sci. (2023) 13:896. 10.3390/educsci13090896

[68] Reeves PM Pun WH Chung KS. Influence of teacher collaboration on job satisfaction and student achievement. Teach Teach Educ. (2017) 67:227–36. 10.1016/j.tate.2017.06.016

[69] Muehlbacher F Hagenauer G Keller MM. Teachers' emotion regulation in the team-taught classroom: insights into teachers' perspectives on how to regulate and communicate emotions with regard to the team teaching partner. Front Educ. (2022) 12:78724. 10.3389/feduc.2022.787224

[70] Taniguchi H Tanaka K. The influences of interpersonal stressors and interpersonal stress coping on depression among teachers. Jpn J Per. (2020) 28:243–46. 10.2132/personality.28.3.1

[71] Carstensen B Klusmann U. Assertiveness and adaptation: prospective teachers' social competence development and its significance for occupational well-being. Br J Educ Psychol. (2021) 91:500–26. 10.1111/bjep.12377 32914428

[72] Tsubono K Ogawa M Maruyama Y. Comparison of primary school teachers' stress responses between pre-pandemic and pandemic periods: a large-scale nationwide survey in Japan. Ind Health. (2022) 22:36261339. 10.2486/indhealth.2022-0147 36261339 PMC10731416

[73] Zhao Y Guo Y Xiao Y Zhu R Sun W Huang W. The effects of online homeschooling on children, parents, and teachers of grades 1-9 during the COVID-19 pandemic. Med Sci Monit. (2020) 26:925591. 10.12659/MSM.925591 32917849 PMC7507793

[74] Aperribai L Cortabarria L Aguirre T Verche E Borges Á. Teacher's physical activity and mental health during lockdown due to the COVID-2019 pandemic. Front Psychol. (2020) 11:577886. 10.3389/fpsyg.2020.577886 33262727 PMC7685995

[75] Yao Y Xu J. Occupational stress of elementary school teachers after eased COVID-19 restrictions: a qualitative study from China. Front Psychol. (2023) 14:1183100. 10.3389/fpsyg.2023.1183100 37303886 PMC10248454

[76] Liu Y Cui Q Liu Y Zhang J Zhou M Ali T. Countermeasures against economic crisis from COVID-19 pandemic in China: an analysis of effectiveness and trade-offs. Struct Chang Econ Dyn. (2021) 59:482–95. 10.1016/j.strueco.2021.09.017 35317308 PMC8490069

[77] Iwamoto Y Miyakawa D Ohtake F. Introduction to the special issue “the impacts of COVID-19 on the Japanese economy”. Jpn Econ Rev. (2021) 72:329–31. 10.1007/s42973-021-00082-y PMC8200313 34149294

